# Neutral Effect of Increased Dairy Product Intake, as Part of a Lifestyle Modification Program, on Cardiometabolic Health in Adolescent Girls With Overweight/Obesity: A Secondary Analysis From a Randomized Controlled Trial

**DOI:** 10.3389/fnut.2021.673589

**Published:** 2021-05-21

**Authors:** Lauren E. Skelly, Erin N. Barbour-Tuck, Nigel Kurgan, Melissa Calleja, Panagiota Klentrou, Bareket Falk, Andrea R. Josse

**Affiliations:** ^1^School of Kinesiology and Health Science, Faculty of Health, York University, Toronto, ON, Canada; ^2^Department of Kinesiology, Faculty of Applied Health Sciences, Brock University, St. Catharines, ON, Canada; ^3^Centre for Bone and Muscle Health, Faculty of Applied Health Sciences, Brock University, St. Catharines, ON, Canada

**Keywords:** dairy products, obesity, adolescent health, cardiometabolic risk, weight management program, exercise, nutrition

## Abstract

**Background:** The presence of obesity and some cardiometabolic disease risk factors in childhood and adolescence track into adulthood. Intake of dairy products has been shown to be inversely related to adiposity and cardiometabolic variables in youth. However, limited research has examined cardiometabolic disease risk factors following increased dairy product consumption as part of a lifestyle modification intervention in youth with overweight/obesity. This secondary analysis aimed to determine whether 12 weeks of increased dairy consumption, as part of a lifestyle modification program, affects cardiometabolic variables in adolescent females (range: 10–18 years) with overweight/obesity (BMI > 85th centile).

**Methods:** Participants were randomized into two groups: higher dairy intake (RDa; four servings/day [to reflect previous Canada's Food Guide recommendations]; *n* = 23) or low dairy intake (LDa; 0–2 servings/day; *n* = 23). Both RDa and LDa participated in a 12-week, eucaloric, lifestyle modification intervention consisting of exercise training, and nutritional counseling. Adiposity (percent body fat [%BF]), dietary intake, and measures of cardiometabolic health were measured pre- and post-intervention.

**Results:** There were no significant changes over time within groups or differences over time between groups for triglycerides (TG), total cholesterol (TC), high-density lipoprotein cholesterol (HDL), TC/HDL ratio, low-density lipoprotein cholesterol (LDL), glucose, insulin, homeostatic model assessment of insulin resistance, adiponectin, and tumor necrosis factor alpha (TNF-α) (main effects of time and interactions, *p* > 0.05). Leptin decreased over the 12-week lifestyle intervention in both groups (main effect of time, *p* = 0.02). After combining the groups (*n* = 46), significant correlations were found between change in %BF and change in some cardiometabolic variables (HDL [*r* = −0.40], TC/HDL ratio [*r* = 0.42], LDL [*r* = 0.36], and TNF-α [*r* = 0.35], *p* < 0.05). After controlling for change in dairy product intake, the correlations were unchanged.

**Conclusion:** Our findings demonstrate that increased dairy product consumption, as part of a lifestyle modification, weight management intervention, had a neutral effect on cardiometabolic disease risk factors in adolescent females with overweight/obesity. Change in dairy product intake did not influence the relationships between change in adiposity and change in cardiometabolic variables. Future research designed to primarily assess the effect of increased dairy product consumption on cardiometabolic disease risk factors in this population is warranted.

**Clinical Trial Registration:** Clinicaltrials.gov; NCT#02581813.

## Introduction

The rates of child and adolescent overweight and obesity (OW/OB) remain a global public health concern. The seriousness of this health concern is, in part, because an obese phenotype beginning in childhood tends to remain stable into adulthood (1–3). While it is rare for children and adolescents to be diagnosed with cardiovascular disease or to exhibit multiple criteria of the metabolic syndrome, the presence of some cardiometabolic disease risk factors, as well as OW/OB, in childhood and adolescence track into adulthood and are early antecedents of future diseases (4, 5). In 2013, 27% of Canadian youth aged 3–19 years old had OW/OB (6). In 2007–2009, only 2% of Canadian youth 10–18 years had the metabolic syndrome, but 38% had at least one metabolic syndrome risk factor (7). Thus, interventions that aim to reduce OW/OB and cardiometabolic disease risk factors should begin early to improve child and adolescent health as well as to help prevent chronic cardiometabolic diseases in adulthood.

Currently, no single pediatric cardiometabolic risk assessment standard exists, but variables used to indicate risk often include one or more of glucose metabolism (fasting glucose, insulin, or insulin resistance, homeostatic model assessment of insulin resistance [HOMA-IR]), cholesterol (total cholesterol [TC]; high-density lipoproteins [HDL]), triglycerides (TG), and waist circumference (WC) (8–10). Global cardiometabolic risk is another framework used to discuss one's comprehensive cardiometabolic risk, which includes the presence of traditional risk factors of cardiovascular disease (e.g., hypertension and high cholesterol), the metabolic syndrome criteria (e.g., waist circumference, and triglycerides), and inflammatory cytokines and adipokines (e.g., tumor necrosis factor alpha [TNF-α], low adiponectin) (11, 12). Global cardiometabolic risk has been supported for use in children and adolescents in whom one or more of the components of the metabolic syndrome may be present without the overt syndrome, or without traditional cardiovascular disease risk factors (12), however, its use for youth is not yet widely accepted. Nonetheless, the measurement of several cardiometabolic markers, including and in addition to traditional risk factors, can improve our understanding of how lifestyle interventions improve overall cardiometabolic health in pediatric populations.

Lifestyle interventions that incorporate healthy dietary changes can improve cardiometabolic outcomes in youth (13). Dairy products are rich sources of macronutrients and micronutrients, including protein, calcium, and vitamin D, and have been important dietary components of successful weight management interventions in adults, in part, because they promote favorable body composition and musculoskeletal changes (14–17). However, the effect of dairy product consumption on cardiometabolic health is mixed. Meta-analyses demonstrate that dairy product consumption is inversely associated with risk of OW/OB in youth (18, 19), and a cross-sectional multi-center study reported that higher dairy consumption was inversely associated with an improved cardiovascular risk score in adolescent females (20). In contrast, a review of evidence from randomized control trials (RCTs) suggests that dairy product consumption has a neutral effect on cardiometabolic health outcomes, including blood lipids (21). In terms of inflammation, while one systematic review concluded that dairy product consumption has no adverse effects on inflammatory markers (22), a more recent review of clinical trials, primarily conducted in adults, across different weight statuses concluded that dairy product consumption has a predominantly anti-inflammatory effect, particularly in those with metabolic disorders (23). To date there is a paucity of controlled research studies in adolescents with OW/OB that specifically investigate dairy product consumption and cardiovascular disease risk markers.

Cardiorespiratory fitness and physical activity levels demonstrate beneficial relationships with cardiometabolic disease risk factors in youth (24, 25), and some exercise interventions have improved markers of cardiometabolic health in adolescents with OW/OB (26, 27). Given the mixed findings (i.e., overall benefit or neutral) between dairy consumption and indices of cardiometabolic health and the benefits of dairy (and its components) on growth and development, lifestyle modification interventions including dairy consumption and exercise, that assess cardiometabolic disease risk markers, particularly in youth with OW/OB, are warranted. Our group has recently demonstrated that dairy intake with exercise promoted fat loss in adolescent females with OW/OB (28). Other similar investigations that have assessed the combined effect of both dairy intake and structured exercise training on body composition have been conducted in adolescent males of normal weight (29), and in a mix of males and females of varying weight statuses (30). These investigations demonstrated no benefit of increased dairy consumption on body fat and did not examine markers of cardiometabolic health. As females generally consume fewer dairy products than males (31, 32), and because of the less well-characterized relationships between changes in dairy intake, adiposity and cardiometabolic disease risk factors in youth, the objective of this investigation was to determine the effect of increased dairy product intake, as part of a 12-week diet and exercise weight management intervention, on cardiometabolic disease risk variables, and to assess the relationship between changes in adiposity and these markers in adolescent females with OW/OB.

## Methods

### Participants

This study represents a secondary analysis of cardiometabolic disease risk factors (blood lipids, insulin, glucose, inflammation, adipokines) and their relationship to adiposity (percent body fat [%BF]) from the “*I.D.E.A.L. (Improving Diet, Exercise and Lifestyle) for Adolescents Study*”. The main study was a 12-week randomized, controlled, parallel intervention trial, primarily designed to assess the effect of consuming 4 servings/day of dairy products vs. a low dairy diet (0–2 servings/day) on body composition outcomes (fat mass [FM], lean mass and %BF) in adolescent females with OW/OB (28). The lifestyle modification intervention for weight management also included mixed-mode exercise training and dietary guidance for both intervention groups. Details for the main study in accordance with CONSORT, including primary outcome results and informed assent and consent, have been published elsewhere (28). To be eligible for the study, female participants had to be between 10 and 18 years old, menarcheal, have OW (body mass index [BMI] > 85th−97th percentile) or OB (BMI > 97th percentile) based on World Health Organization criteria, low dairy consumers (≤2 servings/day), and minimally active (≤2 structured exercise sessions/week) (28). Exclusion criteria included any diagnosed illness or disease, diagnosed dairy allergy or lactose intolerance (28). The study was cleared by our Brock University's Biosciences Research Ethics Board (BREB 14-284) and was registered at www.clinicaltrials.gov as NCT#02581813.

### Study Design

As previously described (28, 33), participants were stratified by BMI percentile (overweight or obesity) and randomly assigned using a random number generator to one of three different groups (recommended dairy [RDa], low dairy [LDa], and no-intervention control) using an unblocked random allocation ratio of 2:2:1. The present secondary analysis includes participants from the RDa and LDa groups only, which were randomized equally (1:1; CONSORT diagram, Supplementary Figure 1). The no-intervention control group was not included in this study due to a low sample size for the cardiometabolic outcomes. Forty-six participants (24 in the recommended dairy group [RDa] and 22 in the low dairy group [LDa]) completed the intervention in terms of the primary outcome of body composition. Blood samples were not obtained from one participant in RDa and thus, cardiometabolic variables were not measured. Also, one participant from LDa withdrew from the study at week 8 (i.e., did not complete) due to an unrelated injury but a blood sample was obtained upon exit and her data were included in this analysis. Thus, the final analysis for this secondary investigation included 23 participants in RDa and 23 participants in LDa (Supplementary Figure 1). Both the RDa and LDa participants underwent the intervention and were provided with individualized dietary advice by a registered dietitian, and an individualized exercise program by certified personal trainers over the 12 weeks. Additionally, RDa was provided with and given guidance on how to consume 4 servings of mixed dairy foods/day, while LDa was instructed to maintain their habitually low dairy intakes. Measurements were conducted and blood samples were obtained at week 0 and 12 for both groups.

### Body Composition and Anthropometrics

At the beginning and end of the 12 weeks, body mass was measured on a standard scale (Digital Physician Scale, Rice Lake Weighing Systems, Rice Lake, WI) wearing light clothing and no shoes. Stature was measured using a stadiometer (Seca 213 Portable Stadiometer, CME Corp., Warwick, RI) with no shoes. WC measures were taken at the level of the umbilicus using a standard, retractable, non-metallic tape measure under the clothing. BMI (kg/m^2^) was calculated using the mass and height from the scale and stadiometer, respectively. Adiposity (i.e., FM and %BF) was assessed using the BodyMetrix device (BMX; BodyMetrix™ System, BX-2000, IntelaMetrix, Inc., Livermore, CA), as previously described (28).

### Exercise Intervention

The exercise intervention consisted of three, guided, 60–90-min mixed-exercise sessions per week throughout the 12-week intervention. Briefly, each session included an aerobic exercise workout, combined with either a resistance or plyometric exercise workout. The aerobic exercise portion of each exercise session was completed on a stationary bike, treadmill, elliptical machine, or a rowing ergometer. The resistance exercise portion consisted of 4–5 exercises that encompassed the whole body, including seated row, chest press, leg curl, and leg press. The plyometric exercise consisted of 4–5 exercises for which participants completed 3 sets of 8–15 jumps per exercise, for a total of 96–225 jumps per workout. Further, details have been reported elsewhere (28).

### Dietary Intervention

Energy requirements and recommended dietary intakes were determined for each participant (28). During the intervention, energy intake was not restricted. Participants met with a registered dietitian 5 times throughout the 12 weeks who instructed them on how to meet nutrient and energy requirements in a healthy way. The recommendations provided by the registered dietitian focused primarily on general healthy eating patterns. For example, advice to both groups included minimizing/avoiding processed foods, higher fat foods, sugar-sweetened beverages, pastries and candy/confection, and replacing with fruit, vegetables, lean meats, meat alternatives, and foods higher in fiber and whole grains. The RDa group was also provided with 4 servings/day of dairy foods in accordance with the previous recommendation in the 2007–2018 Canada's Food Guide (34). This included two cups of 1% milk (one chocolate and one white), 2 × 100 g of 0 or 2% fat Greek yogurt (fat content varied by flavor), and 42 g of cheddar or marble full-fat cheese. Participants in the RDa group were further counseled on how to incorporate the provided dairy foods into their diets without increasing their total energy intake. As reported in our previous publication, general observations from the RDa group's food records suggested that unhealthy snacks and breakfast items (e.g., cookies, high-sugar iced-coffee drinks, slushies, buttered toast, juice boxes, chips and candy) were most often replaced with the provided milk, yogurt and cheese (28). The LDa group was encouraged to maintain their low dairy intakes (0–2 servings/day) and to consume non-dairy protein sources. The LDa group was also asked to avoid calcium-fortified beverages and juices (e.g., soy, almond, rice, and orange/fruit juices).

Participants recorded their dietary intakes with 7-day food records at weeks 0 and 12, and 3-day food records at weeks 2, 4, and 8. Food records were analyzed with *ESHA* (Food Processor SQL, ESHA Research, Salem, OR.). To measure adherence with the dairy protocol, the consumption of daily servings of dairy products was assessed from the weeks 4, 8, and 12 food records. RDa and LDa participants were considered adherent if they consumed ≥3 or ≤2 dairy product servings/day, respectively. As previously reported (28), all RDa and LDa participants were adherent based on these criteria.

### Blood Samples for Cardiometabolic Variables

Overnight (10–12 h) fasted, rested blood samples were collected between the hours of 08:00 and 10:00 at weeks 0 and 12. Blood was collected in standard vacutainer tubes and centrifuged at ≤1300 RCF (g) for 15 min. Serum and plasma were aliquoted into 0.5 ml cryovials and stored at −80°C until analysis. The Cholestech LDX System (A&D Medical, Mississauga, ON, Canada) was used to analyze TC, TG, HDL, low-density lipoprotein (LDL) and glucose from serum. Insulin, leptin and TNF-α were analyzed in duplicate using a microbead multiplex assay kit (Human bone magnetic bead panel, cat# HBNMAG-51K-08, EMD Millipore Corp. Burlington, MA, USA). The average inter- and intra-assay coefficients of variation were 6.0 and 5.7% for insulin, 6.1 and 5.6% for leptin, and 3.7 and 4.9% for TNF-α. Adiponectin was analyzed in duplicate using an ELISA kit (EZHADP-61K Human Adiponectin, EMD Millipore Corp. Burlington, MA, USA), and the inter- and intra-assay coefficients of variation were 2.6 and 1.4%, respectively. HOMA-IR was calculated as the product of glucose (mmol/L) and insulin (mU/L) divided by 22.5 (35).

### Statistical Analysis

Results are presented as mean ± standard deviation (SD). Normality was assessed for each variable and each group separately at weeks 0 and 12 by analyzing skewness and kurtosis and the Shapiro Wilks test. Variables that were not normally distributed were log- or square root-transformed, improving normality. Missing data or invalid (e.g., below the detection limit) data for cardiometabolic disease risk variables at only one time point (week 0 or 12) were replaced with the corresponding week 0 or week 12 data for that participant, creating no change over time. If a value was below the detection limit for both week 0 and 12, the detection limit was inserted. One participant's fasting insulin values were removed from the analysis due to invalid data. Overall, these treatments impacted ~4% of the cardiometabolic data points analyzed (Supplementary Figure 1).

To provide descriptive context of how the lifestyle intervention impacted the participants used in this secondary analysis, previously reported data (height, body mass, BMI, WC, %BF, FM, energy intake, dietary macronutrient, calcium and vitamin D intake, and daily dairy servings) were described and analyzed again. Because of the sample utilized herein, these analyses are slightly different from those previously reported (28, 33).

Changes over time and group differences for height, body mass, WC, cardiometabolic variables, energy intake, dietary macronutrient, dairy servings, and dairy-related micronutrient intakes were assessed using repeated measures analysis of variance (RM-ANOVA), with group (RDa and LDa) as the between-subject factor and time (week 0 and 12) as the within-subject factor. Changes over time and group differences for FM, and %BF were assessed using repeated measures analysis of covariance (RM-ANCOVA) with change in body mass as the covariate.

To further explore the relationships between changes in cardiometabolic disease risk factors and changes in adiposity, partial correlations between change in %BF and change in cardiometabolic variables were run with and without change in dairy intake as a control variable. Change in %BF was selected as our measure of adiposity for our exploratory correlational analyses because it provides a relative measure of change in adiposity. In our participants, over the course of the 12-week lifestyle intervention, both height and lean mass [reported elsewhere (28)] increased. Thus, it is important to explore associations between change in cardiometabolic variables and change in relative adiposity as this measure considers other changes that occurred over the intervention as well as changes due to growth. Statistical analyses were performed using SPSS (Version 27.0 for Windows, Armonk, NY: IBM Corp.) *P*-values were considered significant at <0.05.

## Results

Baseline descriptive characteristics of the participants in RDa and LDa are shown in Table 1. There were significant increases in height (RDa: 0.4 ± 0.5; LDa: 0.5 ± 0.9 cm) and decreases in waist circumference (RDa: −1.4 ± 4.7; LDa: −2.2 ± 4.7 cm) in both groups over the intervention (main effects for time, *p* < 0.05). There were no changes in body mass (RDa: 0.1 ± 3.4; LDa: −0.5 ± 3.4 kg) or BMI (RDa: −0.1 ± 1.2; LDa: −0.3 ± 1.2 kg/m^2^; main effects for time, *p* > 0.05). There was a significant decrease in %BF in both groups (RDa: −1.7 ± 1.6; LDa: −1.2 ± 1.1 %; main effect for time, *p* < 0.001). FM decreased to a greater extent in RDa (−1.4 ± 2.2 kg) compared to LDa (−1.1 ± 2.0 kg) over the intervention (interaction, *p* = 0.04).

**Table 1:** Baseline descriptive characteristics for the participants in the RDa and LDa groups.

**Variable**	**RDa (*n* = 23)**	**LDa (*n* = 23)**
Age (yrs)	14.6 ± 2.3	14.9 ± 2.3
Height (cm)	163.4 ± 8.0	163.6 ± 5.9
Body mass (kg)	80.3 ± 15.4	79.2 ± 13.4
BMI (kg/m^2^)	30.1 ± 5.2	29.6 ± 5.0
WC (cm)	97.9 ± 13.3	95.3 ± 12.9
%BF (%)	38.2 ± 3.7	36.8 ± 3.8
FM (kg)	31.1 ± 8.3	29.5 ± 7.5

*Data are presented as mean ± standard deviation. BMI, Body mass index; %BF, Percent body fat; FM, Fat mass. Similar values for these variables were previously published in (28)*.

Dietary variables are shown in Table 2. There were no significant changes in energy or fat intake in both groups (main effect for time *p* > 0.05). Carbohydrate intake decreased over the intervention in both groups (main effect of time, *p* = 0.04). Dietary protein, calcium and vitamin D intake, and daily dairy servings increased to a greater extent in RDa compared to LDa (interactions *p* < 0.001).

**Table 2:** Select daily dietary intake variables for the participants in the RDa and LDa groups at week 0 to week 12 of the intervention.

**Variable**	**RDa (*****n*** **=** **23)**		**LDa (*****n*** **=** **23)**		**RMANOVA**		
	**Wk0**	**Wk12**	**Wk0**	**Wk12**	**Group effect**	**Time effect**	**Interaction**
Energy (kcal)	1656 ± 437	1731 ± 364	1621 ± 428	1450 ± 251	0.07	0.51	0.10
Protein (g)	65 ± 18	89 ± 13	66 ± 16	71 ± 11	**0.03**	** <0.001**	**0.001**
Carbohydrate (g)	205 ± 64	198 ± 46	202 ± 61	169 ± 36	0.21	**0.04**	0.19
Fat^†^ (g)	67 ± 17	67 ± 24	63 ± 20	58 ± 15	0.11	0.52	0.54
Calcium^†^ (mg)	697 ± 277	1325 ± 177	534 ± 253	489 ± 178	** <0.001**	** <0.001**	** <0.001**
Vitamin D^‡^ (mcg)	2.4 ± 1.6	5.6 ± 1.3	1.6 ± 1.0	1.8 ± 1.2	** <0.001**	** <0.001**	** <0.001**
Dairy Foods^‡^ (svg/day)	1.3 ± 0.6	3.7 ± 0.7	0.9 ± 0.8	0.4 ± 0.4	** <0.001**	**0.001**	** <0.001**

*Data are presented as mean ± standard deviation. Significant at p < 0.05. Group, Time and Interaction p-values are presented from the RMANOVA (p < 0.05 presented in bold). Analyses were run with log- or ^‡^square root-transformed variables. Similar values for these variables were previously published in (28, 33)*.

Cardiometabolic variables are shown in Table 3. There were no significant changes over time or differences over time between groups for TG, TC, HDL, TC/HDL ratio, LDL, glucose, insulin, HOMA-IR, adiponectin, and TNF-α (main effects of time and interactions, *p* > 0.05). Leptin decreased over the 12-week intervention in both groups (main effect of time, *p* = 0.02). There were main effects of group for insulin and HOMA-IR (*p* = 0.04 and *p* = 0.03, respectively), reflecting higher values for RDa compared to LDa at both time points (Table 3).

**Table 3:** Cardiometabolic disease risk variables for the participants in the RDa and LDa groups at week 0 to week 12 of the intervention.

**Variable**	**RDa (*****n*** **=** **23)**		**LDa (*****n*** **=** **23)**		**RMANOVA**		
	**Wk0**	**Wk12**	**Wk0**	**Wk12**	**Group effect**	**Time effect**	**Interaction**
TG^‡^ (mmol/L)	1.3 ± 0.7	1.3 ± 0.6	1.1 ± 0.5	1.1 ± 0.5	0.28	0.91	0.62
TC (mmol/L)	4.2 ± 0.7	4.1 ± 0.6	4.1 ± 0.6	4.1 ± 0.7	0.84	0.84	0.53
HDL^‡^ (mmol/L)	1.2 ± 0.3	1.2 ± 0.4	1.2 ± 0.4	1.2 ± 0.3	0.43	0.61	0.81
TC/HDL^†^ (mmol/L)	4.1 ± 2.0	4.0 ± 1.7	3.6 ± 1.1	3.5 ± 1.1	0.39	0.68	0.93
LDL (mmol/L)	2.4 ± 0.7	2.4 ± 0.7	2.4 ± 0.6	2.4 ± 0.7	0.99	0.99	0.63
Glucose^†^ (mmol/L)	4.8 ± 0.3	4.8 ± 0.3	4.7 ± 0.3	4.6 ± 0.3	0.20	0.94	0.24
Insulin^†^* (pg/ml)	1120 ± 626	1045 ± 567	807 ± 579	882 ± 765	**0.04**	0.63	0.70
HOMA-IR^†^*	6.8 ± 3.9	6.3 ± 3.3	4.9 ± 4.0	5.3 ± 5.0	**0.03**	0.64	0.83
Leptin^†^ (pg/ml)	31124 ± 14964	27502 ± 14520	28191 ± 15282	27006 ± 18250	0.55	**0.02**	0.51
Adiponectin^†^ (ng/ml)	14963 ± 6631	13533 ± 6470	17211 ± 11763	15941 ± 9412	0.49	0.053	0.43
TNF-α^†^ (pg/ml)	3.9 ± 0.9	4.1 ± 1.6	3.7 ± 0.9	3.8 ± 1.1	0.29	0.36	0.99

Data are presented as mean ± standard deviation. Significant at p < 0.05. Group, Time and Interaction p-values are presented from the RMANOVA (p < 0.05 presented in bold). Analyses were run with ^†^log- or ^‡^square root-transformed variables. *n = 22 for RDa. Wk0, Week 0; Wk12, Week 12; TG, Triglycerides; TC, Total cholesterol; HDL, High-density lipoprotein; TC/HDL, Total cholesterol/high-density lipoprotein cholesterol ratio; LDL, Low-density lipoprotein; HOMA-IR, Homeostasis model assessment of insulin resistance; TNF-α, Tumor necrosis factor-alpha.

Because we observed no interactions between group and time for the cardiometabolic variables, we collapsed the intervention groups to assess the global relationship between changes in adiposity, using change in %BF, and change in individual cardiometabolic variables. There were moderate correlations between change in adiposity and changes in some cardiometabolic variables. Specifically, change in %BF correlated with change in HDL (*r* = −0.40), TC/HDL ratio (*r* = 0.42), LDL (*r* = 0.36), and TNF-α (*r* = 0.35) (Table 4). Controlling for dairy intake did not further change or strengthen these relationships indicating that dairy intake had no influence on the relationships between change in adiposity and change in cardiometabolic variables.

**Table 4:** Group aggregated (*n* = 46) partial correlations on change in percent body fat and cardiometabolic variables with and without change in dairy product servings as a control variable.

**Variable**	**Change in %BF**		**Change in %BF controlling for change in dairy intake**	
	**r value**	***p*-value**	**r value**	***p*-value**
Change in TG	0.26	0.08	0.25	0.09
Change in TC	0.28	0.06	0.28	0.07
Change in HDL	−0.40	**0.01**	−0.42	**0.004**
Change in TC/HDL	0.42	**0.004**	0.43	**0.003**
Change in LDL	0.36	**0.02**	0.36	**0.02**
Change in Glucose	0.07	0.66	0.10	0.50
Change in Insulin^†^	0.22	0.14	0.19	0.21
Change in HOMA-IR^†^	0.23	0.12	0.21	0.18
Change in Leptin	0.23	0.13	0.21	0.18
Change in Adiponectin	−0.13	0.41	−0.13	0.38
Change in TNF-α	0.35	**0.02**	0.36	**0.02**

*The correlational analyses were performed on non-transformed values. n = 45. p < 0.05 presented in bold. %BF, Percent body fat; TG, Triglycerides; TC, Total cholesterol; HDL, High-density lipoprotein; TC/HDL, Total cholesterol/high-density lipoprotein cholesterol ratio; LDL, Low-density lipoprotein; HOMA-IR, Homeostatic model assessment of insulin resistance; TNF-α, Tumor necrosis factor-alpha*.

## Discussion

The key finding from this secondary analysis is that a higher dairy intake (4 servings/day), consisting of mixed dairy products ranging in fat content, in combination with exercise and dietary counseling, did not affect (adversely or beneficially) cardiometabolic variables in adolescent girls with OW/OB. Despite no effect, this finding extends our previously published results from this intervention study highlighting significant improvements in body composition and markers of bone metabolism with increased dairy consumption (28, 33). Additionally, our previous observation that both RDa and LDa decreased fat mass following the 12-week lifestyle intervention corroborates our finding from the present study that leptin decreased in both groups. We also found favorable associations between changes in adiposity and changes in HDL, TC/HDL ratio, LDL, and TNF-α. While these relationships were not influenced by changes in dairy product consumption, correlations suggest that, through our lifestyle modification intervention (with or without increased dairy intake), reductions in adiposity were favorably associated with changes in some variables that contribute to increased cardiometabolic disease risk.

Our study provides novel data regarding the effect of increased dairy product intake, as part of a lifestyle modification program involving structured exercise training and dietary counseling, on cardiometabolic disease risk markers in adolescent females with OW/OB. Although, we noted that some participants in our study had elevated risk factors, most of them did not present with the metabolic syndrome, according to the International Diabetes Federation consensus definition of metabolic syndrome for children and adolescents (10). This may be a reason for the lack of observed change or group differences in cardiometabolic variables with the intervention. Indeed, while 89% of our participants surpassed even the adult female cut-off for WC (80 cm) and 41% had low age-specific HDL (<1.03 mmol/L ≤ 15 years; <1.29 mmol/L ≥ 16 years), only 15% had TG over cut-offs (≥ 1.7 mmol/L), and none surpassed fasting glucose cut-offs (≥ 5.6 mmol/L) (10). Many of the positive effects of dairy on cardiometabolic disease risk outcomes, and relationships therein are found in those with OW/OB who also have several pre-existing risk factors or diagnosed metabolic disorders (23, 36). Thus, our participants may have been relatively “too healthy” to see significant changes.

Many studies assessing the effect of dairy on blood lipids, and/or inflammatory markers have been carried out in adult populations, and are summarized in narrative and systematic reviews, which have suggested that dairy product intake has neutral to beneficial effects (21–23, 37, 38). The most recent of these reviews included 27 RCTs in adults without severe inflammatory disorders and concluded that dairy product and/or dairy protein consumption had no adverse effects and possibly beneficial effects on low-grade systemic inflammation; a finding that was most commonly observed in adults with OW/OB (37). None of these reviews specifically assessed these relationships in youth with/without exercise.

A few RCTs have assessed increased dairy intake on markers of cardiometabolic health in pediatric (i.e., 5–18 years) populations with OW/OB (39–42). St-Onge et al. (40) implemented 16 weeks of healthy diet counseling supplemented with either high-milk (944 ml/d) or low-milk (236 ml/d) consumption in children (mean age 9.4 ± 0.8 years) with OW and found no beneficial changes in blood lipids, fasting glucose, or insulin in the high-milk group relative to the low-milk group. A longer 3 year RCT in younger children (mean age 5.6 ± 0.5 years) with OB by Kelishadi et al. (39) compared changes in cardiovascular disease risk factors in three groups: those consuming a dairy-rich diet (>800 mg ca/day) plus healthy lifestyle counseling, those on a calorie-restricted diet plus healthy lifestyle counseling, and those given only the healthy lifestyle counseling. Following the initial 6 months, TG, HDL fasting insulin and HOMA-IR were improved in all groups, but this improvement was maintained at 1 year only in the group consuming a dairy-rich diet. After 3 years, these markers of cardiometabolic health appeared similar across the 3 groups. Thus, like our study, these studies (39, 40) report little to no benefit of increased dairy intake on cardiometabolic variables. Although, the participants in our study differed substantially in age and maturation from those in St-Onge et al. (40) and Kelishadi et al. (39), all participants were of pediatric age, and were undergoing substantial growth, development and maturation, and thus dairy-related improvements in markers of cardiometabolic disease risk may have been masked by these concurrent physiological changes. Interestingly, St-Onge et al. (40) found that 16 weeks of high-milk consumption improved insulin area under the curve following an oral glucose tolerance test to a greater extent than low-milk consumption. Measuring cardiometabolic variables in a challenged, postprandial state, may be more informative from a disease risk standpoint than measuring under static, fasting conditions (43). Indeed, postprandial measures of glucose and TG in adults show stronger associations with cardiovascular events relative to fasting measures (44, 45), and this may be the case in youth too. Thus, to assess whether dairy product consumption improves cardiometabolic health it may also be important for future studies in adolescents with OW/OB to assess these cardiometabolic outcomes in a postprandial or challenged state.

Several intervention studies have included structured exercise training and assessed cardiometabolic outcomes in adolescents with OW/OB, and some have combined nutritional counseling (without a focus on dairy) with supervised exercise training (27). Two 4-month RCTs involving combined exercise training and nutritional education have reported improvements in fasting glucose (46), HOMA-IR and markers of inflammation (47) in adolescents with OW/OB. In addition, a 1 year family-based weight management program consisting of exercise, nutritional education, and behavior modification improved body composition, TC, fasting insulin and HOMA-IR relative to a control group in youth with OB (48). Despite the similar study designs reflecting our LDa group, there were several differences between these studies compared to ours that could account for the different results including a longer study duration and higher initial body fat levels. Also, it is important to note that our main intervention was not designed with cardiometabolic disease risk factors as the primary endpoint, and thus, we may have been underpowered. Future studies should be undertaken with adequate power to specifically detect changes in cardiometabolic disease risk factors with and without dairy in adolescent females and males undergoing lifestyle modification for obesity management.

Although, our study was conducted in the context of energy balance producing weight maintenance, weight loss in youth is associated with improvements in some cardiometabolic outcomes (49). To this effect, there are also lifestyle interventions in youth that have focused on weight loss and have improved cardiometabolic disease risk factors (50, 51). For example, a 12-week diet and exercise intervention induced weight and fat loss, and improved blood lipids, fasting insulin and HOMA-IR in children (range: 6–11 years) with obesity (50). In adult populations, our previous research in premenopausal females with OW/OB undergoing a 4-month diet and exercise intervention also demonstrated significant beneficial changes in cardiometabolic variables (i.e., blood lipids, inflammation) with weight and fat loss (14). Dairy intake in this study did not affect these results. Thus, weight loss (with fat loss) may be an important driver for change in cardiometabolic disease risk factors, and dairy product intake, in lifestyle modification programs, adds no further benefit. In the present investigation, the lack of improvement in indices of cardiometabolic health may be related to the lack of overall weight loss, despite improvements in adiposity in both groups. This notion is corroborated by previous research in youth with OB (52, 53) and could be further explored in the context of weight loss and dairy consumption.

In our study, participants in both groups were counseled to eat a healthy diet, and while both groups improved body composition, the high dairy group saw greater reductions in fat mass (28). While this reduction did not translate to significant improvements in markers of cardiometabolic risk, our correlations suggest that a reduction in adiposity was favorably associated with changes in some cardiometabolic variables, specifically HDL, TC/HDL ratio, LDL, and TNF-α. Similarly, Lira at al. (54) conducted a 1-year single-arm exercise, nutrition, and psychological intervention study that induced weight and fat loss but showed no significant improvements in inflammation in adolescents with OB. The authors also observed significant positive correlations between visceral fat (measured by ultrasonography) and inflammatory markers (54). Therefore, in both our study and the study by Lira et al. (54), adiposity was positively associated with inflammation during a lifestyle intervention where adiposity improved over time (i.e., fat mass and %BF decreased) without significant improvements in inflammation. Of note, in our study, this association was found in the absence of weight loss (but with significant fat loss) and independent of dairy consumption.

Throughout puberty, the trajectories of most cardiometabolic disease risk factors (e.g., TG, HDL, LDL, insulin, HOMA-IR) are curvilinear (8, 55). Changes in these variables during this time may make it more difficult to identify significant improvements in these variables (56). Indeed, in accordance with the patterns of pubertal change for these cardiometabolic disease risk factors, the International Diabetes Federation cardiometabolic risk cut-offs are age-specific, grouping together 10 to 15-year-olds and 16- to 18-year-olds (10). As such, observations of “no change” in cardiometabolic variables in our study may not actually be due to a lack of a response to the intervention, *per-se*, but rather to the variability in the physiological levels of these factors and the natural progression of these pubertal changes (56). For example, because younger adolescents, aged 10–13 years, undergo a period of transient insulin resistance indicated by an increasing HOMA-IR (55), the absence of change in fasting insulin and HOMA-IR over our intervention, could indicate a positive adaptation. While we only recruited participants who were menarcheal, our participants' ages spanned 10–19 years (by the end of the intervention), and there can be up to 5 years of variability in biological maturation at a single chronological age, especially during times of peak pubertal change (at ~11–13 years old in females) (57). Indeed the participant heterogeneity across the cardiometabolic variables was large, and we did not have a reliable measure of maturation [e.g., Tanner staging or years from menarche (57)] to ensure that sexual and somatic maturation was similar between groups. Thus, the ability of our intervention to significantly improve markers of cardiometabolic disease risk may have been masked by different trajectories of pubertal development and the variability therein. Nonetheless, intensive multicomponent interventions that work to improve healthy lifestyle habits remain the recommendation for adolescents with OB (58) and are effective at reducing adiposity and some cardiometabolic outcomes (13, 59). As such, it is important to continue to design intervention studies with the purpose of improving body composition (specifically decreasing body fat) and decreasing cardiometabolic disease risk within the context of lifestyle modification and weight management in this population that can account for the inherent variability during the adolescent period.

In summary, our study demonstrated that, compared to a low dairy diet, the addition of a variety of dairy foods (4 servings/day) to a diet and exercise, lifestyle modification program for weight management did not differentially change markers of cardiometabolic disease in adolescent females with OW/OB. Favorable associations were found between changes in adiposity and changes in cardiometabolic disease risk factors, but these were not altered after controlling for changes in dairy consumption. Our previous findings from this intervention demonstrate the positive effect of dairy consumption on musculoskeletal health, body composition and nutrient intake (28, 33); and, while the current findings demonstrate no additional benefit (or harm) of dairy on indices of cardiometabolic health, we assert that dairy foods should still be included as part of a healthy diet for adolescent girls with OW/OB during this critical time of growth and development. Future research designed to primarily assess the effect of increased dairy product consumption on cardiometabolic disease risk factors in this population is warranted.

## Data Availability Statement

The raw data supporting the findings of this study are available from the corresponding author, Andrea R. Josse, upon reasonable request.

## Ethics Statement

The studies involving human participants were reviewed and approved by Brock University's Biosciences Research Ethics Board (BREB 14-284). Written informed consent to participate in this study was also provided by the participants' legal guardian/next of kin.

## Author Contributions

PK, BF, and AJ: study design conceptualization. LS, EB-T, NK, MC, PK, BF, and AJ: data analysis, interpretation, and drafting and revising manuscript. All authors approved of the final version of this manuscript.

## Conflict of Interest

AJ, BF, and PK report grants for this research from Dairy Farmers of Canada, US National Dairy Council (Dairy Management Inc.), non-financial support from Danone and Parmalat during the conduct of the study; AJ reports personal fees from Dairy Farmers of Canada Grant Review Board outside the submitted work. LS and EB-T report some salary support from the US National Dairy Council grant. The remaining authors declare that the research was conducted in the absence of any commercial or financial relationships that could be construed as a potential conflict of interest.

## References

[1] Anderson LN Lebovic G Hamilton J Hanley AJ McCrindle BW Maguire JL. Body mass index, waist circumference, and the clustering of cardiometabolic risk factors in early childhood. Paediatr Perinat Epidemiol. (2016) 30:160–70. 10.1111/ppe.12268 26645704

[2] Barbour-Tuck E Erlandson M Muhajarine N Foulds H Baxter-Jones A. Influence of childhood and adolescent fat development on fat mass accrual during emerging adulthood: a 20-year longitudinal study. Obesity (Silver Spring). (2018) 26:613–20. 10.1002/oby.22111 29377622

[3] Herman KM Craig CL Gauvin L Katzmarzyk PT. Tracking of obesity and physical activity from childhood to adulthood: the Physical Activity Longitudinal Study. Int J Pediatr Obes. (2009) 4:281–8. 10.3109/17477160802596171 19922043

[4] Camhi SM Katzmarzyk PT. Tracking of cardiometabolic risk factor clustering from childhood to adulthood. Int J Pediatr Obes. (2010) 5:122–9. 10.3109/17477160903111763 19593726

[5] Kelly AS Steinberger J Jacobs DR Hong CP Moran A Sinaiko AR. Predicting cardiovascular risk in young adulthood from the metabolic syndrome, its component risk factors, and a cluster score in childhood. Int J Pediatr Obes. (2011) 6:e283–9. 10.3109/17477166.2010.528765 21070100 PMC3684392

[6] Rodd C Sharma AK. Recent trends in the prevalence of overweight and obesity among Canadian children. CMAJ. (2016) 188:E313–20. 10.1503/cmaj.150854 27160875 PMC5026530

[7] MacPherson M de Groh M Loukine L Prud'homme D Dubois L. Prevalence of metabolic syndrome and its risk factors in Canadian children and adolescents: Canadian Health Measures Survey Cycle 1 (2007-2009) and Cycle 2 (2009-2011). Health Promot Chronic Dis Prev Can. (2016) 36:32–40. 10.24095/hpcdp.36.2.03 26878492 PMC4910427

[8] Cook S Auinger P Huang TT-K. Growth curves for cardio-metabolic risk factors in children and adolescents. J Pediatr. (2009) 155:S6.e15–26. 10.1016/j.jpeds.2009.04.051 19732566 PMC2789447

[9] Kamel M Smith BT Wahi G Carsley S Birken CS Anderson LN. Continuous cardiometabolic risk score definitions in early childhood: a scoping review. Obes Rev. (2018) 19:1688–99. 10.1111/obr.12748 30223304

[10] Zimmet P Alberti K George MM Kaufman F Tajima N Silink M. The metabolic syndrome in children and adolescents - an IDF consensus report. Pediatr Diabetes. (2007) 8:299–306. 10.1111/j.1399-5448.2007.00271.x 17850473

[11] Calabro P Yeh ETH. Intra-abdominal adiposity, inflammation, and cardiovascular risk: new insight into global cardiometabolic risk. Curr Hypertens Rep. (2008) 10:32–8. 10.1007/s11906-008-0008-z 18367024

[12] Despres JP Lemieux I Bergeron J Pibarot P Mathieu P Larose E. Abdominal obesity and the metabolic syndrome: contribution to global cardiometabolic risk. Arterioscler Thromb Vasc Biol. (2008) 28:1039–49. 10.1161/ATVBAHA.107.159228 18356555

[13] Ho M Garnett SP Baur L Burrows T Stewart L Neve M. Effectiveness of lifestyle interventions in child obesity: systematic review with meta-analysis. Pediatrics. (2012) 130:e1647–71. 10.1542/peds.2012-1176 23166346

[14] Josse AR Atkinson SA Tarnopolsky MA Phillips SM. Increased consumption of dairy foods and protein during diet- and exercise-induced weight loss promotes fat mass loss and lean mass gain in overweight and obese premenopausal women. J Nutr. (2011) 141:1626–34. 10.3945/jn.111.141028 21775530 PMC3159052

[15] Zemel MB Richards J Mathis S Milstead A Gebhardt L Silva E. Dairy augmentation of total and central fat loss in obese subjects. Int J Obes (Lond). (2005) 29:391–7. 10.1038/sj.ijo.0802880 15672113

[16] Stonehouse W Wycherley T Luscombe-Marsh N Taylor P Brinkworth G Riley M. Dairy intake enhances body weight and composition changes during energy restriction in 18-50-year-old adults-a meta-analysis of randomized controlled trials. Nutrients. (2016) 8:394. 10.3390/nu8070394 27376321 PMC4963870

[17] Thorning TK Bertram HC Bonjour JP de Groot L Dupont D Feeney E. Whole dairy matrix or single nutrients in assessment of health effects: current evidence and knowledge gaps. Am J Clin Nutr. (2017) 105:1033–45. 10.3945/ajcn.116.151548 28404576

[18] Wang W Wu Y Zhang D. Association of dairy products consumption with risk of obesity in children and adults: a meta-analysis of mainly cross-sectional studies. Ann Epidemiol. (2016) 26:870–82.e2. 10.1016/j.annepidem.2016.09.005 27756684

[19] Lu L Xun P Wan Y He K Cai W. Long-term association between dairy consumption and risk of childhood obesity: a systematic review and meta-analysis of prospective cohort studies. Eur J Clin Nutr. (2016) 70:414–23. 10.1038/ejcn.2015.226 26862005

[20] Bel-Serrat S Mouratidou T Jiménez-Pavón D Huybrechts I Cuenca-García M Mistura L. Is dairy consumption associated with low cardiovascular disease risk in European adolescents? Results from the HELENA Study. Pediatr Obes. (2014) 9:401–10. 10.1111/j.2047-6310.2013.00187.x 23852857

[21] Drouin-Chartier JP Côté JA Labonté MÈ Brassard D Tessier-Grenier M Desroches S. Comprehensive review of the impact of dairy foods and dairy fat on cardiometabolic risk. Adv Nutr. (2016) 7:1041–51. 10.3945/an.115.011619 28140322 PMC5105034

[22] Labonté MÈ Couture P Richard C Desroches S Lamarche B. Impact of dairy products on biomarkers of inflammation: a systematic review of randomized controlled nutritional intervention studies in overweight and obese adults. Am J Clin Nutr. (2013) 97:706–17. 10.3945/ajcn.112.052217 23446894

[23] Bordoni A Danesi F Dardevet D Dupont D Fernandez AS Gille D. Dairy products and inflammation: A review of the clinical evidence. Crit Rev Food Sci Nutr. (2017) 57:2497–525. 10.1080/10408398.2014.967385 26287637

[24] Skrede T Aadland E Andersen LB Stavnsbo M Anderssen SA Resaland GK. Does cardiorespiratory fitness moderate the prospective association between physical activity and cardiometabolic risk factors in children? Int J Obes (Lond). (2018) 42:1029–38. 10.1038/s41366-018-0108-z 29777236

[25] Ekelund U Anderssen SA Froberg K Sardinha LB Andersen LB Brage S. Independent associations of physical activity and cardiorespiratory fitness with metabolic risk factors in children: the European youth heart study. Diabetologia. (2007) 50:1832–40. 10.1007/s00125-007-0762-5 17641870

[26] Vasconcellos F Seabra A Katzmarzyk PT Kraemer-Aguiar LG Bouskela E Farinatti P. Physical activity in overweight and obese adolescents: systematic review of the effects on physical fitness components and cardiovascular risk factors. Sports Med. (2014) 44:1139–52. 10.1007/s40279-014-0193-7 24743931

[27] Stoner L Rowlands D Morrison A Credeur D Hamlin M Gaffney K. Efficacy of exercise intervention for weight loss in overweight and obese adolescents: meta-analysis and implications. Sports Med. (2016) 46:1737–51. 10.1007/s40279-016-0537-6 27139723

[28] Calleja M Caetano Feitoza N Falk B Klentrou P Ward WE Sullivan PJ. Increased dairy product consumption as part of a diet and exercise weight management program improves body composition in adolescent females with overweight and obesity-A randomized controlled trial. Pediatr Obes. (2020) 15:e12690. 10.1111/ijpo.12690 32602233

[29] Volek JS Gómez AL Scheett TP Sharman MJ French DN Rubin MR. Increasing fluid milk favorably affects bone mineral density responses to resistance training in adolescent boys. J Am Diet Assoc. (2003) 103:1353–6. 10.1016/S0002-8223(03)01073-3 14520257

[30] Lambourne K Washburn RA Lee J Betts JL Thomas DT Smith BK. A 6-month trial of resistance training with milk supplementation in adolescents: effects on body composition. Int J Sport Nutr Exerc Metab. (2013) 23:344–56. 10.1123/ijsnem.23.4.344 23239680

[31] Green BP Turner L Stevenson E Rumbold PLS. Short communication: patterns of dairy consumption in free-living children and adolescents. J Dairy Sci. (2015) 98:3701–5. 10.3168/jds.2014-9161 25795492

[32] Nezami M Segovia-Siapco G Beeson WL Sabaté J. Associations between consumption of dairy foods and anthropometric indicators of health in adolescents. Nutrients. (2016) 8:427. 10.3390/nu8070427 27420094 PMC4963903

[33] Josse AR Ludwa IA Kouvelioti R Calleja M Falk B Ward WE. Dairy product intake decreases bone resorption following a 12-week diet and exercise intervention in overweight and obese adolescent girls. Pediatr Res. (2020) 88:910–6. 10.1038/s41390-020-0834-5 32179870

[34] Health Canada/Government of Canada. Eating Well with Canada's Food Guide 2007. (2011). Available online at: https://www.canada.ca/en/health-canada/services/canada-food-guide/about/history-food-guide/eating-well-with-canada-food-guide-2007.html (accessed January 26, 2021).

[35] Matthews DR Hosker JP Rudenski AS Naylor BA Treacher DF Turner RC. Homeostasis model assessment: insulin resistance and beta-cell function from fasting plasma glucose and insulin concentrations in man. Diabetologia. (1985) 28:412–9. 10.1007/BF00280883 3899825

[36] Pereira MA Jacobs DR Jr Van Horn LV Slattery ML Kartashov AI Ludwig DS. Dairy consumption, obesity, and the insulin resistance syndrome in young adults: the CARDIA study. JAMA. (2002) 287:2081–9. 10.1001/jama.287.16.2081 11966382

[37] Nieman KM Anderson BD Cifelli CJ. The effects of dairy product and dairy protein intake on inflammation: a systematic review of the literature. J Am Coll Nutr. (2020). 10.1080/07315724.2020.1800532. [Epub ahead of print]. 32870744

[38] Ulven SM Holven KB Gil A Rangel-Huerta OD. Milk and dairy product consumption and inflammatory biomarkers: an updated systematic review of randomized clinical trials. Adv Nutr. (2019) 10(suppl_2):S239–50. 10.1093/advances/nmy072 31089732 PMC6518147

[39] Kelishadi R Zemel MB Hashemipour M Hosseini M Mohammadifard N Poursafa P. Can a dairy-rich diet be effective in long-term weight control of young children? J Am Coll Nutr. (2009) 28:601–10. 10.1080/07315724.2009.10719792 20439556

[40] St-Onge MP Goree LL Gower B. High-milk supplementation with healthy diet counseling does not affect weight loss but ameliorates insulin action compared with low-milk supplementation in overweight children. J Nutr. (2009) 139:933–8. 10.3945/jn.108.102079 19321584 PMC2714393

[41] Arnberg K Mølgaard C Michaelsen KF Jensen SM Trolle E Larnkjær A. Skim milk, whey, and casein increase body weight and whey and casein increase the plasma C-peptide concentration in overweight adolescents. J Nutr. (2012) 142:2083–90. 10.3945/jn.112.161208 23077192

[42] Ghayour-Mobarhan M Sahebkar A Vakili R Safarian M Nematy M Lotfian E. Investigation of the effect of high dairy diet on body mass index and body fat in overweight and obese children. Indian J Pediatr. (2009) 76:1145–50. 10.1007/s12098-009-0231-x 20012799

[43] van Ommen B Keijer J Heil SG Kaput J. Challenging homeostasis to define biomarkers for nutrition related health. Mol Nutr Food Res. (2009) 53:795–804. 10.1002/mnfr.200800390 19517455

[44] Cavalot F Petrelli A Traversa M Bonomo K Fiora E Conti M. Postprandial blood glucose is a stronger predictor of cardiovascular events than fasting blood glucose in type 2 diabetes mellitus, particularly in women: lessons from the San Luigi Gonzaga Diabetes Study. J Clin Endocrinol Metab. (2006) 91:813–9. 10.1210/jc.2005-1005 16352690

[45] Bansal S Buring JE Rifai N Mora S Sacks FM Ridker PM. Fasting compared with nonfasting triglycerides and risk of cardiovascular events in women. JAMA. (2007) 298:309–16. 10.1001/jama.298.3.309 17635891

[46] Davis JN Tung A Chak SS Ventura EE Byrd-Williams CE Alexander KE. Aerobic and strength training reduces adiposity in overweight Latina adolescents. Med Sci Sports Exerc. (2009) 41:1494–503. 10.1249/MSS.0b013e31819b6aea 19516150 PMC2836768

[47] Balagopal P George D Patton N Yarandi H Roberts WL Bayne E. Lifestyle-only intervention attenuates the inflammatory state associated with obesity: a randomized controlled study in adolescents. J Pediatr. (2005) 146:342–8. 10.1016/j.jpeds.2004.11.033 15756217

[48] Savoye M Shaw M Dziura J Tamborlane WV Rose P Guandalini C. Effects of a weight management program on body composition and metabolic parameters in overweight children: a randomized controlled trial. JAMA. (2007) 297:2697–704. 10.1001/jama.297.24.2697 17595270

[49] Rajjo T Almasri J Al Nofal A Farah W Alsawas M Ahmed AT. The association of weight loss and cardiometabolic outcomes in obese children: systematic review and meta-regression. J Clin Endocrinol Metab. (2017) 102:758–62. 10.1210/jc.2016-2575 28359092

[50] Shalitin S Ashkenazi-Hoffnung L Yackobovitch-Gavan M Nagelberg N Karni Y Hershkovitz E. Effects of a twelve-week randomized intervention of exercise and/or diet on weight loss and weight maintenance, and other metabolic parameters in obese preadolescent children. Horm Res. (2009) 72:287–301. 10.1159/000245931 19844115

[51] Reinehr T de Sousa G Toschke AM Andler W. Long-term follow-up of cardiovascular disease risk factors in children after an obesity intervention. Am J Clin Nutr. (2006) 84:490–6. 10.1093/ajcn/84.3.490 16960161

[52] Reinehr T Kleber M Toschke AM. Lifestyle intervention in obese children is associated with a decrease of the metabolic syndrome prevalence. Atherosclerosis. (2009) 207:174–80. 10.1016/j.atherosclerosis.2009.03.041 19442975

[53] Browning MG Bean MK Wickham EP Stern M Evans RK. Cardiometabolic and fitness improvements in obese girls who either gained or lost weight during treatment. J Pediatr. (2015) 166:1364–9. 10.1016/j.jpeds.2015.03.011 25890676 PMC4446179

[54] Lira FS Rosa JC Dos Santos RV Venancio DP Carnier J Sanches Pde L. Visceral fat decreased by long-term interdisciplinary lifestyle therapy correlated positively with interleukin-6 and tumor necrosis factor-alpha and negatively with adiponectin levels in obese adolescents. Metabolism. (2011) 60:359–65. 10.1016/j.metabol.2010.02.017 20359719

[55] Shashaj B Luciano R Contoli B Morino GS Spreghini MR Rustico C. Reference ranges of HOMA-IR in normal-weight and obese young Caucasians. Acta Diabetol. (2016) 53:251–60. 10.1007/s00592-015-0782-4 26070771

[56] Reinehr T. Metabolic syndrome in children and adolescents: a critical approach considering the interaction between pubertal stage and insulin resistance. Curr Diab Rep. (2016) 16:8. 10.1007/s11892-015-0695-1 26747052

[57] Cameron N. Assessment of maturation. In: Cameron N, editor. Human Growth and Development. 1st ed: San Diego, CA: Academic Press (2002). p. 363–82. 10.1016/B978-012156651-7/50018-6

[58] US Preventive Services Task Force Grossman DC Bibbins-Domingo K Curry SJ Barry MJ Davidson KW. Screening for obesity in children and adolescents: US preventive services task force recommendation statement. JAMA. (2017) 317:2417–26. 10.1001/jama.2017.6803 28632874

[59] Rajjo T Mohammed K Alsawas M Ahmed AT Farah W Asi N. Treatment of pediatric obesity: an umbrella systematic review. J Clin Endocrinol Metab. (2017) 102:763–75. 10.1210/jc.2016-2574 28359101

