# Supplementary material for: Neutral Effect of Increased Dairy Product Intake, as Part of a Lifestyle Modification Program, on Cardiometabolic Health in Adolescent Girls With Overweight/Obesity: A Secondary Analysis From a Randomized Controlled Trial
Source: Front Nutr. 2021 May 21;8:673589. doi: 10.3389/fnut.2021.673589 (PMC8175852; doi:10.3389/fnut.2021.673589)
Supplement: Supplementary file 1 [file Data_Sheet_1.PDF]

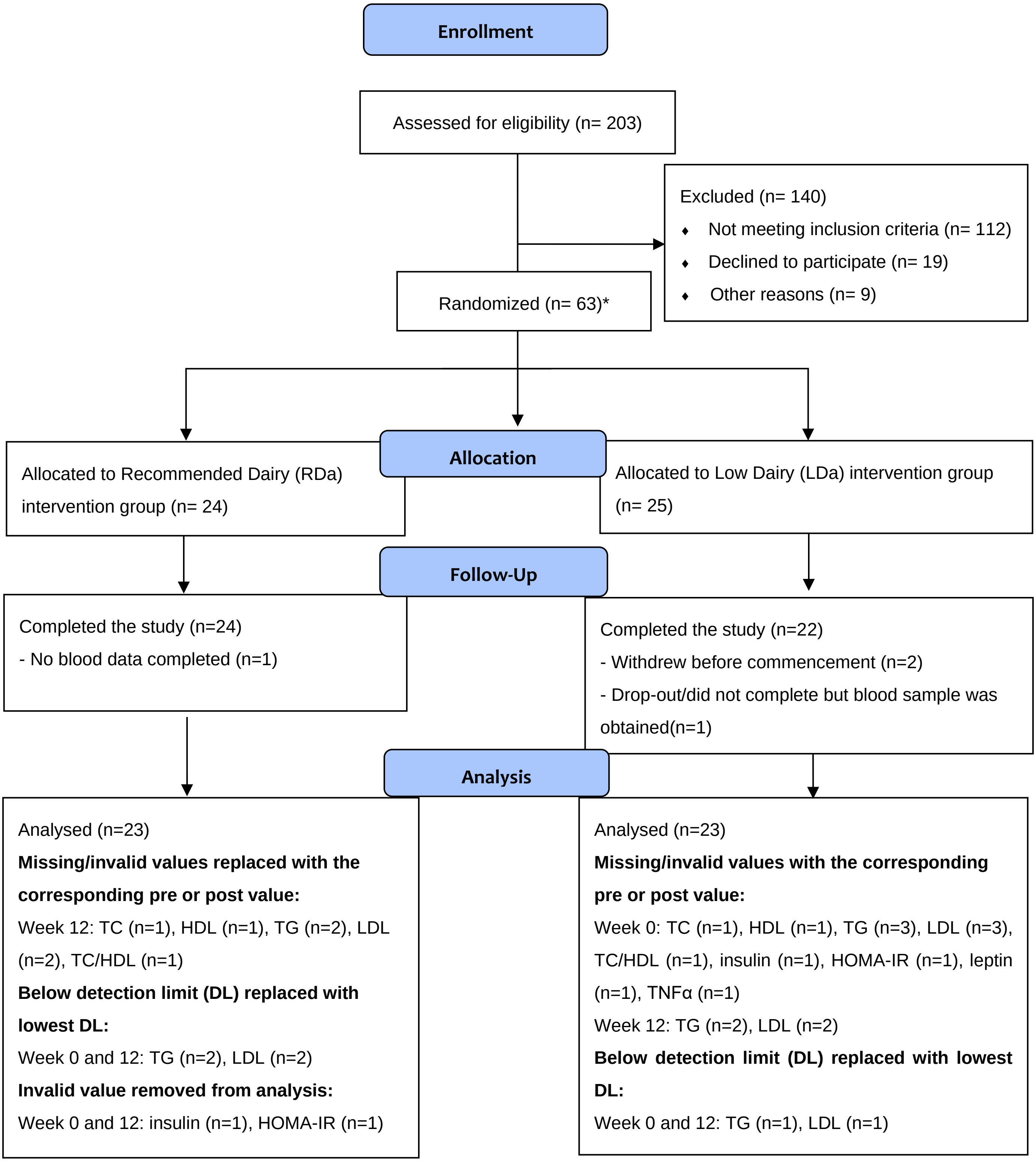

Supplementary Figure 1: Consort Flow Diagram.

*The remaining participants were allocated to a no-intervention control group not reported in this study.
